# Functional application of monkey jack (*Artocarpus lacucha*) pulp extract as a natural preservative: Effects on quality, safety, and storage stability of raw pangasius fish balls

**DOI:** 10.1371/journal.pone.0357592

**Published:** 2026-09-03

**Authors:** Md. Golam Rabby, Md. Nayim Hossain, Maymuna Islam Keya, Md. Ashrafuzzaman Zahid, Rashida Parvin, Md Syduzzaman

**Affiliations:** Department of Nutrition and Food Technology, Jashore University of Science and Technology, Jashore, Bangladesh; Al-Farabi Kazakh National University, KAZAKHSTAN

## Abstract

This study evaluated how Monkey jack pulp extract (MJPE) impacted the raw Pangasius (*Pangasianodon hypophthalmus*) fish balls physicochemical quality, microbiological safety, oxidative, and storage stability while being refrigerated. Fish balls were made with several antioxidants, including control (T0), butylated hydroxytoluene (BHT, T1), ascorbic acid (AA, T2), and MJPE at increasing concentrations (T3 and T4), and kept for 10 days at 4°C. On days 1, 5, and 10, changes in pH, cooking loss, color characteristics (L*, a*, b*, chroma, and hue angle), heme iron content, antioxidant activity (DPPH), lipid oxidation (TBARS), and total viable count (TVC) were assessed. All samples showed quality decrease with increasing storage time. However, antioxidants treated samples showed significantly (*P* < 0.05) higher stability than the control treated samples in every storage day. T4 successfully inhibited pH rise, decreased cooking loss, maintained higher heme iron levels, and preserved color characteristics rather than other treatments. Additionally, T4 showed higher DPPH scavenging capacity (ranged 72.44% to 65.79%), whereas the control showed the lower DPPH value (ranged 56.57% to 33.39%). TBARS value showed the lowest (0.31 to 0.41 mg MDA/kg sample) in T4 and the highest (0.49 to 0.67 mg MDA/kg sample) in the control. MJPE considerably reduced bacterial growth (0.80 to1.68 Log10 CFU/mL) compared to control and synthetic antioxidants treated samples. In terms of keeping the quality and enhancing the shelf life, MJPE demonstrated significantly improved antioxidant and antimicrobial effects compared to BHT and AA (*P* < 0.05). MJPE showed significant potential as a natural preservative for fish-based products by improving their physicochemical, microbiological, and oxidative stability during refrigerated storage.

## Introduction

Pangas (*Pangasianodon hypophthalmus)* is among the fastest-growing fish species in the world [1]. Its proteins, polyunsaturated and omega-3 fatty acids promote heart health, and its easy digestion benefits both patients and children [2]. Fish balls are round-shaped products made by mixing minced fish with other ingredients [3]. Diversification of fisheries products is required to meet consumers increasing need for variety in fish-based diets [4]. Fish balls can be eaten raw or fried, and they have been a common snack in Bangladesh and throughout Asia for many years. Traditionally made from fish species, provide a nutrient-dense, protein-rich alternative (20.8–30.2%) by scraping out the bones and decreasing fishy odor by heating [5]. Despite being a rich source of nutrients, particularly protein, water, and unsaturated fatty acids, fish balls and other fish products are susceptible to lipid oxidation and both enzymatic and non-enzymatic rancidity during storage [5,6]. These lipids oxidation, leading to the formation of primary and secondary oxidation products such as hydroperoxides and malondialdehyde (MDA), which adversely affect flavor, color, and nutritional quality in fish and fish products. Additionally, refrigerated conditions slow microbial growth but do not prevent oxidative reactions, allowing lipid peroxidation to continue over time. This oxidative deterioration contributes significantly to quality loss and shelf-life reduction in fish ball products [7]. The use of different preservation methods and non-thermal technologies to extend food shelf life has become increasingly important due to the rising demand for fresh, high-quality refrigerated foods [8].

In order to delay, slow, or prevent lipid oxidation in fish products, synthetic antioxidants such as BHT, butylated hydroxyanisole (BHA), and AA are normally used [9]. Additionally, antioxidants can be added, mixed directly into mince, or sprayed, coated, or injected into entire muscle portions in meat and fish to decrease microbial deterioration, extend shelf life, and maintain quality in refrigerated fish products [10]. The maximum concentration of synthetic antioxidants permitted is 0.02% on the lipid basis, which is generally effective to stabilize natural foods such as meat, with the potential for very lengthy storage or deep fat frying [11]. Although these compounds are generally recognized as safe within regulated limits, concerns have been raised regarding their potential health effects when consumed in excessive amounts [12]. Previous studies have reported that the excessive use of synthetic antioxidants may be associated with adverse health effects, including toxicity and carcinogenicity [13]. Studies have shown the advantages of using natural antioxidants from a variety of fruits and vegetables or their byproducts to preserve food quality and reduce negative effects, despite current safety concerns about the use of natural preservatives rather than synthetic ones [14,15]. Therefore, there is growing interest in natural alternatives that may offer similar preservative functions with improved consumer acceptance [16]

Monkey jackfruit *(Artocarpus lacucha)* is a native plant species found in North Sumatera, Indonesia [17]. The genus monkey jack, which is part of the Angiosperms division, belongs to the mulberry family (*Moraceae*) and includes 64 recognized species [18]. Drying monkey jackfruit pulp may turn it into a powder while preserving its nutrients. It contains carbohydrates (fructose, glucose, and sucrose), minerals, vitamin C, and β-carotene, which is a precursor to vitamin A and acts as an antioxidant [19]. To date, there is limited direct research on the antioxidant effects of MJPE in fish-based systems or its role in inhibiting lipid oxidation. However, studies on other plant-derived pulps have demonstrated that incorporation of antioxidant-rich materials can significantly reduce lipid oxidation in fish products, as evidenced by decreased peroxide and TBARS values. These effects are primarily attributed to the presence of phenolic compounds, which can donate hydrogen atoms or electrons to neutralize free radicals and interrupt lipid peroxidation chain reactions. Additionally, phenolics may chelate pro-oxidant metal ions, thereby further inhibiting oxidative deterioration and enhancing product stability during storage. Therefore, it is reasonable to hypothesize that MJPE, being rich in bioactive compounds, may exert similar antioxidant effects in fish-based systems [20,21].

Although numerous plant extracts have been investigated for their antioxidant and antimicrobial properties [22], limited studies have explored the application of MJPE in fish-based food systems. MJPE contains diverse bioactive compounds, including phenolics and flavonoids, which have been reported to possess antioxidant and antimicrobial activities [23]. However, to the best of our knowledge, its application as a natural preservative for controlling lipid oxidation and microbial growth in fish products has not been extensively investigated, despite the growing interest in plant-derived natural preservatives for fish preservation [22].

Therefore, the novelty of this study lies in evaluating MJPE as a natural preservative in raw Pangasius fish balls, with a comprehensive assessment of physicochemical quality, microbial safety, and storage stability under refrigerated conditions. The study further compares the effectiveness of MJPE with conventional preservatives BHT and AA in reducing lipid oxidation and microbial growth during storage.

## Materials and methods

### Collection of the monkey jack fruits and formulation of MJPE

Fresh ripened monkey jack fruits were collected from a local shop in Jashore, Bangladesh. Once the peel and seeds had been removed, the pulp was collected and dried in an oven at 55°C for 15 h (all experiments involving the fruit were performed in accordance with relevant institutional, national, and international guidelines). The dried pulp was then finely blended using a GAZI blender (230 VAC-50 Hz, Bangladesh) and sieved by a 500-micron (ASTM No. 35) mesh to obtain a uniform fine powder [24]. Soxhlet extraction was performed using 85% ethanol as the solvent, selected for its efficiency in extracting polar and moderately polar bioactive compounds such as phenolics and flavonoids. Briefly, 150 g of finely powdered sample was mixed with solvent at a 1:4 (w/v) ratio and extracted in a Soxhlet apparatus for 20 h [25]. The powdered sample was placed in a cellulose thimble within the extraction chamber. The solvent in the bottom flask was heated to reflux, vaporized, condensed, and continuously percolated through the sample, allowing efficient extraction through repeated cycles. The extraction duration was selected to ensure maximum recovery of bioactive compounds while maintaining compound stability. After 20 h of extraction, the mixture was filtered by use of Whatman No. 1 filter paper (Karami et al., 2015). The filtrate was then concentrated operating a rotary vacuum evaporator (RE-A2000, Scitek, China) at 55°C for 2 h until all the ethanol evaporated, producing a semi-liquid extract. The extract was further dried in an oven for 20 h to obtain the MJPE insemi solid form, which was stored at –20°C until further use [17].

### Collection of pangasius fish and preparation of pangasius fish ball

The fish (10 kg) was gathered from a nearby marketplace in Jashore, Bangladesh, and stored at −20°C until further use. The fish was properly prepared by removing the scales, fins, and internal organs and then thoroughly washed [5]. After being frozen, the fish meat was roughly smashed for 1 min by use of a grinder (Sonifer meat grinder). The minced fish meat was subsequently divided into four groups. Each 20 g fish ball was formulated with the following basic ingredients: 90% Pangasius fish, 4% ice water, 4.5% corn starch, and 1.5% salt. In treatments T1 (BHT), T2 (AA), T3 and T4 (MJPE) were added at 0.02%, 0.05%, 0.1% and 0.3%, respectively, while T0 served as the control without antioxidant (Table 1). The concentrations of MJPE used in this study were selected based on preliminary experiments and relevant literature to achieve effective antioxidant and antimicrobial activity. The selected levels also enabled comparison with conventional preservatives such as BHT and AA, which are commonly used within regulated limits.

**Table 1:** The formulation of fresh fish balls with the addition of several antioxidants.

Ingredients (%)	Treatments				
	T0	T1	T2	T3	T4
Fish meat	90	90	90	90	90
Salt	1.5	1.5	1.5	1.5	1.5
Corn starch	4.5	4.5	4.5	4.5	4.5
Ice water	4.0	4.0	4.0	4.0	4.0
BHT	–	0.02	–	–	–
AA	–	–	0.05	–	–
0.1% MJPE	–	–	–	0.1	–
0.3% MJPE	–	–	–	–	0.3
Total	100.0	100.02	100.05	100.1	100.3

All the ingredients were placed in a bowl and mixed with each antioxidant (using gloves) for 10 min to obtain a uniform batter for different treatments. The fish ball mixture was molded into round balls weighing 20 g each. After that, the fish balls were vacuum-packed and properly kept at 4°C. Samples were taken on days 1, 5, and 10 to evaluate their functional properties, chemical characteristics and microbial properties [26].

### pH evaluation

The pH was measured by homogenizing 3 g of fish ball sample with 27 mL of distilled water. The mixture was allowed to stand for 30 min at room temperature and then filtered. The pH of the resulting filtrate was measured using a calibrated digital pH meter (HI2211, Hanna Instruments, USA) equipped with an electrode probe (HI1131). Each sample was analyzed in triplicate, and the average value was reported [27,28].

### Cooking loss

Fish balls were cooked in a water bath (preheated) at 100°C ± 2 for 15 min on each side. Cooking loss was determined by measuring the weight difference of the samples before and after cooking. The cooking loss was calculated using Equation (1) as described by [29,30].


$$\begin{array}{c} \text{Cooking loss }(\%)\;=\;\frac{\text{Weight of uncooked chicken meatballs}-\text{Weight of cooked chicken meatballs}}{\text{Weight of uncooked chicken meatballs}}\;\text{1}\text{0}\text{0}\% \end{array}$$ (1)

### Color measurement

A colorimeter (BCM-110, made in China) was used to determine the color of the fish ball sample. The color assessment consists of chroma hue angle, lightness (L), redness (a), and yellowness (b). At room temperature, the colorimeter was calibrated using the standard white plate (Y = 89.2; x = 0.921; y = 0.783) immediately before conducting the meatball colorimetry test [31,32]. The hue angle and chroma values were calculated using the following formulas, Eqs. (2) and (3):


$$\begin{array}{c} \text{Chroma}=\;\sqrt{a^{\text{2}}+b^{\text{2}}} \end{array}$$ (2)

$$\begin{array}{c} \text{Hue angle value }={\text{tan}}^{-\text{1}}(\frac{b}{a}) \end{array}$$ (3)

### Heme iron

The procedure outlined by [33] was used with some adjustments to determine the fish balls heme iron concentration: To determine the heme iron content, 2 g of the fish balls sample was placed in a tube containing 10 mL of acidified acetone solution. The tube was sealed tightly and left in the dark for 1 h at room temperature. The combination was then filtered through Whatman GFA glass filter paper, and a spectrophotometer was used to measure the absorbance at 640 nm. The heme iron content was calculated by using Eq. (4) and Eq. (5), assuming all pigments were in the form of hematin.


$$\begin{array}{c} \text{Whole pigment }(\text{mg}/\text{kg})\;=\text{ Absorbance }\times \text{ 680} \end{array}$$ (4)

$$\begin{array}{c} \text{Heme iron }(\text{mg}/\text{kg})\;=\;\frac{\text{Whole pigment }(\text{mg}/\text{kg})\;\times \;\text{8}.\text{82}}{\text{100}} \end{array}$$ (5)

### DPPH (2, 2-diphenyl-1-picrylhydrazyl)

Following a few minor modifications, the method described by Thaipong et al., 2006 [34] was used to assess the radical scavenging activity of DPPH. For the DPPH assay, 150 μL of the fish ball filtrate sample was mixed with 2850 μL of 24 mM DPPH solution. The mixture solution was allowed the dark condition for 30 min to ensure complete reaction. A spectrophotometer was used to detect the absorbance at 515 nm following incubation. Equation (6) was then used to calculate the DPPH radical scavenging activity.

%DPPH radical scavenging activity


$$\begin{array}{c} \%\text{ DPPH radical scavenging activity }=\;\frac{\text{Absorbance of control}-\text{ Absorbance of sample }}{\text{Absorbanceof control }}\times \text{1}\text{0}\text{0}\% \end{array}$$ (6)

### Lipid oxidation

The TBARS values for fish ball samples were calculated using the outline given by [35], with a few minor modifications. To homogenize, 27 mL of perchloric acid (3.86%) were mixed with 3 g of the fish ball. Run the USA-made DWB (D160) homogenizer for at least 20 sec to achieve optimal homogeneity. After being kept for 60 min at 4°C, the homogenized samples were centrifuged for 15 min at 3000 rpm using an 80−2 Electronic Centrifuge. The supernatant was filtered out using Whatman No. 1 filter paper. After adding 2 mL of thiobarbituric acid (TBA) to 2 mL of filtrate, the entire mixture was allowed to stand at room temperature without light for 15 h. Spectrophotometry was then used to determine the absorbance for these samples at 531 nm (Cary 60 UV-Vis, Agilent Technologies Inc., Seoul, Korea).

### Microbial test

Each treatments microbiological analysis has been carried out using the technique outlined by [36]. 20 g of the fish ball sample was safely transferred to a sterile stomacher bag and homogenized in 180 mL of sterile saline for 2 min using a stomacher (BagMixer 400 VW, Interscience Co.). 1 mL of the solution from each serial dilution was injected onto the plate count once the 1:10 dilutions were finished. Next, dilutions up to 10^^-1^ should be made successively. To determine the number of microorganisms being investigated, the final diluted fish ball samples (0.2 mL) were dipped in culture media; the total viable count was conducted at 37°C for 24 h.

### Statistical analysis

The experiment was conducted using four treatments with three replications and evaluated at three storage times. All analyses were performed in triplicate, and results are expressed as mean ± standard error. Statistical analysis was carried out using IBM SPSS Statistics (version 26). One-way analysis of variance (ANOVA) was applied to evaluate the effect of treatments at each storage interval separately. When significant differences were observed (p < 0.05), Duncan’s multiple range test was used to compare mean values among treatments. All experiments were conducted under similar conditions, and samples were analyzed in a randomized manner to minimize experimental bias.

## Results and discussions

### Effects of different types of antioxidants on the pH of fresh fish balls at refrigerated storage

The pH value usually describes the acidity and alkalinity levels, which indicate how fresh fish and meat-derived products are [37]. The various antioxidants effect on the pH of fresh fish balls during refrigerated storage (1, 5, and 10 days) is presented in Table 2.

**Table 2:** Effects of different types of antioxidants on the pH of fresh fish balls at refrigerated storage.

Storage day	T0	T1	T2	T3	T4	SEM
1	6.65^Ca^	6.32^Cb^	6.24^Cc^	6.08 Cd	5.82^Ce^	0.02
5	6.82^Ba^	6.53^Bb^	6.36^Bc^	6.23^Bd^	5.92^Be^	0.02
10	6.97^Aa^	6.65^Ab^	6.52^Ac^	6.38^Ad^	6.16^Ae^	0.01
SEM	0.01	0.01	0.02	0.01	0.01	

a–eMean values in the same row with different letters as superscript exhibited significant differences (*P* < 0.05).

A–CMean values in the same column with different letters as superscript exhibited significant differences (*P* < 0.05).

T0: Control; T1: added 0.02% BHT, T2: added 0.05% AA, T3: added 0.1% MJPE, T4: added 0.3% MJPE.

SEM: Standard error of the mean.

Overall, all treatments showed a significant (*P* < 0.05) increase in pH values with storage duration. The increase in pH during storage is often linked to microbial degradation of proteins, which produces basic compounds such as ammonia and amines, signaling spoilage and quality deterioration [38]. At every storage interval, T0 (control) sample consistently showed the highest pH values. On the other hand, during storage, the pH values of the antioxidant-treated samples were noticeably lower than those of the control. T4 (0.3% MJPE) had the lowest pH values on all storage days. Although they had fewer of an impact than MJPE, BHT (T1) and AA (T2) both considerably lowered pH in comparison to the control. *Pangasius hypophthalmus* fish balls were preserved using polyphenol extracts from mangosteen peels, the pH was lower than with other treatments [26]. The outcomes of moringa and lavender extracts as natural antioxidants in tilapia fish burgers were comparable to our study [39]. During cold storage, garlic peel extract is added to anchovy fish patties showed the lower pH value rather than other treatments [40]. Fish balls with natural additions, ascorbate palmitoyl, and oat phenolic compounds displayed a reduced pH value during cold storage [35,41]. The spoilage bacteria may alter the physicochemical characteristics and enhance the buildup of ammonia and trimethylamine throughout the storage period, so raising the pH level [42]. MJPE contains organic acids and antioxidants, which may be the reasons for keeping the pH level stable in the fresh fish ball at refrigerated storage.

### Effects of different types of antioxidants on the cooking loss of fresh fish balls at refrigerated storage

Fish and animal items lost moisture and fat while cooking, as indicated by cooking loss [43]. Table 3 shows the cooking loss of fresh fish balls prepared with various antioxidants alongside control samples.

**Table 3:** Effects of different types of antioxidants on the cooking loss of fresh fish balls at refrigerated storage.

Storage Day	T0	T1	T2	T3	T4	SEM
1	6.39^Ca^	5.09^Cb^	4.63^Cc^	4.07 Cd	2.05^Ce^	0.04
5	8.47^Ba^	6.30^Bb^	5.82^Bc^	5.21^Bd^	3.16^Be^	0.05
10	12.90^Aa^	8.11^Ab^	7.07^Ac^	6.19^Ad^	4.28^Ae^	0.03
SEM	0.03	0.04	0.06	0.02	0.05	

a–eMean values in the same row with different letters as superscript exhibited significant differences (*P* < 0.05).

A–CMean values in the same column with different letters as superscript exhibited significant differences (*P* < 0.05).

T0: Control; T1: added 0.02% BHT, T2: added 0.05% AA, T3: added 0.1% MJPE, T4: added 0.3% MJPE.

SEM: Standard error of the mean.

In both cases, cooking loss increased considerably (*P* < 0.05) with storage duration, suggesting a gradual decline in water-holding capacity during refrigeration. At every storage time, the control sample (T0) showed the highest cooking loss, which increased significantly between day 1 and day 10. On the other hand, throughout storage, all antioxidant-treated samples showed noticeably less cooking loss than the control. MJPE-incorporated samples (T3 and T4) were the most successful in reducing cooking loss among the treatments; T4 (0.3% MJPE) consistently recorded the lowest values across all storage days. AA (T2) and BHT (T1) also considerably decreased cooking loss in comparison to T0; however, their effects were not as strong as those of MJPE. Carp Fish Patties When combined with various plant extracts, the cooking loss was reduced during frozen storage [44]. Surimi fish balls treated with leaf extracts from Perilla (*Perilla frutescens*) displayed the pertinent outcomes [45]. The findings of this investigation were consistent with other research which found a non-significant increase in cooking loss in beef patties treated with clove extract [46]. Because cooking loss was crucial to preserving nutritional status, juiciness, and tenderness [47]. Fish balls treated with MJPE containing antioxidants were less affected during the storage period because of decreased cooking loss.

### Effects of different types of antioxidants on the color attributes of fresh fish balls at refrigerated storage

Color is one of the numerous variables that affect the acceptance of food by consumers. Consumer approval of fish products is highly influenced by their color, including lightness, redness, and yellowness [48]. Meats natural colors are weakened by the oxidation of lipids, and the addition of non-meat ingredients such fillers and meat extenders may also be the cause of color changes [49,50]. The effects of several antioxidants on the color characteristics (L*, a*, b*, chroma, and hue angle) of fresh fish balls during refrigeration are summarized in Table 4.

**Table 4:** Effects of different types of antioxidants on the color attributes of fresh fish balls at refrigerated storage.

	Storage Day	T0	T1	T2	T3	T4	SEM
Lightness(L*)	1	64.60^Ae^	70.87^Ad^	74.28^Ac^	78.90^Ab^	84.51^Aa^	0.97
	5	56.88^Bd^	65.57^Bc^	65.59^Bc^	71.02^Bb^	77.24^Ba^	1.14
	10	54.72^Bc^	60.94^Bb^	60.23^Cb^	63.79^Cb^	70.69^Ca^	0.91
	SEM	1.46	1.38	1.16	0.84	0.47	
Redness(a*)	1	6.40^Ad^	6.55^Ad^	7.10^Ac^	8.21^Ab^	9.43^Aa^	0.06
	5	5.23^Be^	6.04^Bd^	6.67^Bc^	7.76^Bb^	8.96^Ba^	0.03
	10	4.97^Ce^	5.70 Cd	6.41^Cc^	7.43^Cb^	8.46^Ca^	0.02
	SEM	0.04	0.02	0.05	0.04	0.03	
Yellowness(b*)	1	40.02^Ca^	26.33^Cb^	28.82^Cb^	21.90^Bc^	19.79^Bd^	0.81
	5	41.32^Ba^	27.37^Bb^	29.29^Bb^	22.94B^c^	20.24^Bd^	0.65
	10	44.03^Aa^	29.06^Ab^	30.97^Ac^	25.14^Ad^	22.98^Ae^	0.55
	SEM	0.38	1.16	0.68	0.40	0.44	
Chroma(C*)	1	36.47^Ac^	40.63^Ab^	41.62^Ab^	43.26^Ab^	48.00^Aa^	0.94
	5	31.11^Bc^	35.61^Bb^	36.70^Bb^	38.84^Bb^	42.29^Ba^	0.78
	10	26.06^Cc^	31.67^Cb^	32.41^Cb^	33.67^Cb^	37.24^Ca^	0.93
	SEM	0.70	0.70	1.02	0.80	1.13	
Hue angel(h^o^)	1	69.22^Ca^	60.45^Cb^	62.23^Bb^	58.25^Cb^	51.05^Cc^	0.97
	5	76.16^Ba^	65.56^Bb^	68.73^Bb^	62.55^Bc^	56.87^Bd^	1.01
	10	83.33^Aa^	72.87^Ab^	75.58^Ab^	71.02^Ab^	64.24^Ac^	2.49
	SEM	1.90	1.38	2.16	1.07	1.53	

a–eMean values in the same row with different letters as superscript exhibited significant differences (*P* < 0.05).

A–CMean values in the same column with different letters as superscript exhibited significant differences (*P* < 0.05).

T0: Control; T1: added 0.02% BHT, T2: added 0.05% AA, T3: added 0.1% MJPE, T4: added 0.3% MJPE.

SEM: Standard error of the mean.

All treatments showed a significant (*P* < 0.05) decrease in color quality as storage went on, suggesting oxidative alterations and pigment degradation linked to refrigerated storage. Due to the lack of antioxidant defense against oxidative discoloration, the control samples (T0) showed the most noticeable decline in color parameters. All treatments showed a significant drop in lightness (L*) values with storage time; however, samples treated with antioxidants, especially those containing MJPE (T3 and T4), maintained significantly greater L* values than the control. Fish balls made with varying amounts of tea powder had shown higher lightness values than control samples [51]. Fish products made with caffeic acid had significantly lower lightness values than the control [52]. The relatively reduced lightness values of the rainbow fish croquettes made with the brewing dill extract were consistent with our current investigation [26]. During storage, redness (a*) and yellowness (b*) also decreased, with T0 showing the highest losses. On the other hand, samples treated with antioxidants, particularly 0.3% MJPE (T4), showed more stable b* values and higher a* values, suggesting that the natural color of fish balls was better preserved. Potential uses of natural pigment in fish products showed the better redness and reduced amount of yellowness value [53]. Dragon fruit peel as dietary fiber and antioxidant on chicken nuggets showed the similar outcome as we got [54]. Higher redness and lower yellowness values were observed in the quality attributes of spent hen meat patties using kiwi and pineapple extracts [55]. Clove extract on cooked beef patties at refrigerated storage showed the similar results as we get [56]. Antioxidant-treated samples had significantly higher chroma values than the control, although chroma (C*), a measure of color intensity, gradually declined with storage time. MJPE-treated samples showed the strongest effect, indicating improved color saturation and stability. In the same way, hue angle (h°) increased with storage time in all treatments, suggesting a trend toward discoloration; however, the increase was much smaller in samples treated with antioxidants, especially T3 and T4, indicating delayed color degradation. The combined effects of adding turmeric powder and cooking techniques are showed the similar results [57]. Similar outcomes were obtained when Perilla (leaf extracts) was applied to the surimi fish balls [45]. However, antioxidant-treated samples, particularly those containing MJPE (T3 and T4), exhibited greater stability in L*, a*, and b* values compared to the control, suggesting effective inhibition of oxidative discoloration. The preservation of redness and smaller changes in hue angle indicate that MJPE may protect heme pigments and delay lipid oxidation–related color degradation. Overall, MJPE-treated samples demonstrated significantly improved color stability during refrigerated storage (*P* < 0.05), highlighting the role of natural antioxidants in maintaining visual quality. These findings suggest that the bioactive compounds present in MJPE effectively inhibited oxidative processes and preserved color attributes throughout storage.

### Effects of different types of antioxidants on the heme iron content (mg heme iron/kg of sample) of fresh fish balls at refrigerated storage

Animal products are a good source of iron in the form of heme iron. During food product processing or storage, heme iron may be liberated from the porphyrin ring structure, and free ferrous iron may then initiate the oxidation process [58]. The body needs iron for a number of metabolic functions, including storage, electron transmission, and oxygen transport. Iron is also essential for the development of the central nervous system, cell division, and genetic material repair [25]. The impact of antioxidants on the heme iron concentration (mg/kg) of fresh fish balls during refrigeration is shown in Table 5.

**Table 5:** Effects of different types of antioxidants on the heme iron content (mg heme iron/kg of sample) of fresh fish balls at refrigerated storage.

Storage Day	T0	T1	T2	T3	T4	SEM
1	1.50^Ac^	1.64^Ad^	1.54^Ac^	1.70^Ab^	1.84^Aa^	0.03
5	1.34^Bc^	1.54^Bb^	1.42^Bb^	1.52^Bb^	1.70^Ba^	0.03
10	1.24^Cc^	1.42^Ca^	1.32^Bb^	1.38^Ba^	1.48^Ca^	0.03
SEM	0.03	0.02	0.03	0.04	0.02	

a–eMean values in the same row with different letters as superscript exhibited significant differences (*P* < 0.05).

A–CMean values in the same column with different letters as superscript exhibited significant differences (*P* < 0.05).

T0: Control; T1: added 0.02% BHT, T2: added 0.05% AA, T3: added 0.1% MJPE, T4: added 0.3% MJPE.

SEM: Standard error of the mean.

Heme iron content decreased significantly (*P* < 0.05) in all treatments during storage, indicating progressive oxidative degradation of heme pigments under refrigerated conditions. The highest reduction was observed in the control (T0), suggesting higher susceptibility to oxidation in the absence of antioxidant protection. In contrast, antioxidant-treated samples retained higher heme iron levels throughout storage, with MJPE-incorporated treatments (T3 and T4) showing the highest stability. This preservation of heme iron may be attributed to reduced oxidative degradation of heme pigments in the presence of antioxidant compounds. Overall, the findings show that adding antioxidants, especially the highest concentrations of MJPE, effectively postponed the breakdown of heme iron and improved the oxidative stability of fresh fish balls while they were being refrigerated. Fish balls containing moringa leaf powder and fish balls containing turmeric powder from pangas (*Pangasianodon hypophthalmus*) produced comparable results [5,57]. These findings indicate that MJPE effectively delayed heme pigment oxidation and contributed to improved oxidative stability of fish balls during refrigerated storage. However, no conclusions can be drawn regarding the nutritional bioavailability or dietary significance of heme iron based on the present study.

### Effects of different types of antioxidants on the DPPH of fresh fish balls at refrigerated storage

The antioxidant capacity of natural preservatives and food shelf life are related to DPPH readings. By evaluating free radical scavenging activity, DPPH is a metric that demonstrates the antioxidant capacity [59]. The impact of various antioxidants on the DPPH radical scavenging activity of fresh fish balls during refrigeration is shown in Table 6.

**Table 6:** Effects of different types of antioxidants on the DPPH (%) of fresh fish balls at refrigerated storage.

Storage Day	T0	T1	T2	T3	T4	SEM
1	56.57^Ac^	59.46^Ac^	64.90^Ab^	68.11^Aa^	72.44^Aa^	1.79
5	44.34^Bd^	46.54^Bc^	51.46^Bb^	57.63^Bb^	67.26^Ba^	2.00
10	33.39 Cd	41.21^Cc^	44.21^Bc^	52.77^Cb^	65.79^Ba^	0.98
SEM	1.02	1.17	2.73	1.50	1.12	

a–dMean values in the same row with different letters as superscript exhibited significant differences (*P* < 0.05).

A–CMean values in the same column with different letters as superscript exhibited significant differences (*P* < 0.05).

T0: Control; T1: added 0.02% BHT, T2: added 0.05% AA, T3: added 0.1% MJPE, T4: added 0.3% MJPE.

SEM: Standard error of the mean.

As storage time extended, DPPH activity significantly decreased (*P* < 0.05) in all treatments, suggesting a progressive loss of antioxidant potential under refrigeration. Due to the lack of additional antioxidant protection, the control sample (T0) continuously showed the lowest DPPH levels at all storage intervals, with a notable decline from day 1 to day 10. On the other hand, during storage, samples treated with antioxidants showed noticeably more DPPH scavenging activity. MJPE-incorporated samples (T3 and T4) showed the best antioxidant activity across the treatments; T4 (0.3% MJPE) had the highest DPPH values throughout all storage days. Although they were less effective than MJPE, BHT (T1) and AA (T2) both markedly increased DPPH activity in comparison to the control. Higher concentrations of bioactive phenolic compounds that contribute to long-term radical scavenging activity are suggested by MJPE’s improved performance. The antioxidant potential of fresh fish balls was found to be significantly altered by both antioxidant type and storage length, as indicated by different superscript letters within rows and columns that show significant differences (*P* < 0.05) among treatments and storage durations. Overall, the results show that adding antioxidants, especially MJPE at higher concentrations, successfully enhanced and extended the antioxidant stability of fish balls during chilled storage. The DPPH scavenging activity observed in MJPE-treated samples was comparable to that reported for other plant-derived antioxidants. For example, mango peel extract and turmeric powder have been reported to exhibit strong antioxidant capacities in similar food systems, while perilla leaf extract showed comparable trends in surimi-based products [45,57,60]. These findings indicate that MJPE possesses antioxidant activity within the range of commonly used natural extracts.

### Effects of different types of antioxidants on the TBARS (mg MDA/kg sample) of fresh fish balls at refrigerated storage

TBARS value is the principal indicator that defines the food quality. It is the main sign of rancidity and lipid oxidation in food products [50]. Although having a high concentration of biologically important proteins, meat and fish are extremely susceptible to oxidation [61]. The impacts of different antioxidants on lipid oxidation in fresh fish balls during refrigerated storage, determined by TBARS values (mg MDA/kg sample), are presented in Table 7.

**Table 7:** Effects of different types of antioxidants on the TBARS (mg MDA/kg sample) of fresh fish balls at refrigerated storage.

Storage Day	T0	T1	T2	T3	T4	SEM
1	0.49^Ca^	0.43^Bb^	0.45^Cb^	0.36^Bc^	0.31^Bd^	0.01
5	0.58^Ba^	0.54^Ab^	0.51^Bb^	0.43^Ac^	0.36^Bd^	0.01
10	0.67^Aa^	0.58^Ab^	0.56^Ab^	0.46^Ab^	0.41^Ad^	0.02
SEM	0.01	0.01	0.02	0.01	0.01	

a–dMean values in the same row with different letters as superscript exhibited significant differences (*P* < 0.05).

A–CMean values in the same column with different letters as superscript exhibited significant differences (*P* < 0.05).

T0: Control; T1: added 0.02% BHT, T2: added 0.05% AA, T3: added 0.1% MJPE, T4: added 0.3% MJPE.

SEM: Standard error of the mean.

TBARS values increased significantly (*P* < 0.05) during storage across all treatments, indicating progressive lipid oxidation under refrigerated conditions. However, the extent and rate of this increase varied notably among treatments, with the control (T0) exhibiting the most rapid rise, reflecting higher susceptibility to oxidative deterioration in the absence of antioxidant protection. In contrast, all antioxidant-treated samples showed significantly lower TBARS values throughout storage, demonstrating their effectiveness in inhibiting lipid oxidation. Among the treatments, MJPE-incorporated samples (T3 and T4) were the most effective, with T4 (0.3% MJPE) consistently exhibiting the lowest TBARS values at all storage intervals. This indicates that higher concentrations of MJPE provided higher protection against oxidative degradation. Although synthetic antioxidants such as BHT (T1) and AA (T2) also significantly reduced TBARS formation compared to the control, their inhibitory effects were less pronounced than those observed in MJPE-treated samples. The slower progression of TBARS values in MJPE treated samples suggests delayed oxidative kinetics, which is essential for maintaining product quality during refrigerated storage. Furthermore, the relatively lower TBARS levels in these samples indicate improved oxidative stability, which may contribute to the extended shelf life of the fish balls. The effects of thyme, rosemary, and basil extracts on the mackerel balls showed the lower TBARS value [62]. Fishballs made from silver barb (*Barbonymus gonionotus*) using yanang (*Tiliacora triandra*) leaf extract have shown reduced lipid oxidation [63]. Overall, these findings demonstrate that MJPE effectively retards lipid peroxidation and plays a significant role in preserving the quality of fish balls during refrigerated storage.

### Effects of different types of antioxidants on total bacteria count (Log 10 CFU/mL) of fresh fish balls at refrigerated storage

Table 8 shows the total viable count (TVC) of fresh fill balls samples on various storage days.

**Table 8:** Effects of different types of antioxidants on total bacteria count (Log10 CFU/mL) of fresh fish balls at refrigerated storage.

Storage Day	T0	T1	T2	T3	T4	SEM
1	1.22^Ca^	1.12^Cb^	1.12^Cb^	0.96^Bc^	0.80 Cd	0.12
5	1.94^Ba^	1.57^Bb^	1.63^Bb^	1.47^Ac^	1.26^Bd^	0.01
10	2.23^Aa^	1.98^Ab^	2.05^Ab^	1.94^Ab^	1.68^Ac^	0.04
SEM	0.05	0.08	0.06	0.15	0.09	

a–dMean values in the same row with different letters as superscript exhibited significant differences (*P* < 0.05).

A–CMean values in the same column with different letters as superscript exhibited significant differences (*P* < 0.05).

T0: Control; T1: added 0.02% BHT, T2: added 0.05% AA, T3: added 0.1% MJPE, T4: added 0.3% MJPE.

SEM: Standard error of the mean.

All treatments showed a significant (*P* < 0.05) increase in microbial load with storage duration, which is consistent with typical microbial growth under refrigeration. However, depending on the antioxidant used, the rate of growth varied significantly between sessions. Throughout the storage period, the control sample (T0) had the highest bacterial counts, with a noticeable rise from day 1 to day 10. On the other hand, during matching storage days, all antioxidant-treated samples displayed considerably lower total bacterial counts than the control, suggesting that the addition of antioxidants helped to postpone microbial expansion. MJPE-added samples (T3 and T4) were the most successful in inhibiting bacterial growth among the treatments; T4 (0.3% MJPE) consistently recorded the lowest bacterial counts across all storage times. Although their antimicrobial effects were not as strong as those seen for MJPE treatments, BHT (T1) and AA (T2) similarly decreased bacterial growth in comparison to the control. Products developed from tilapia fish (*Oreochromis niloticus*) using natural extracts were shown to be similar to our findings in frozen storage time [64]. Sesame and cinnamon extract in fresh poultry meatballs at refrigerated storage showed the lower TVC value [58]. Overall, the results suggest that MJPE, particularly at higher concentration, is effective in controlling microbial growth and extending shelf life of fresh fish balls during refrigerated storage. Total viable count (TVC) was used as a general indicator of microbial quality and spoilage in the present study. Although specific spoilage organisms and pathogenic bacteria could provide more detailed insights into microbial safety, TVC is widely accepted for assessing overall microbial load in fish products.

## Conclusion

The results of this investigation showed MJPE is a useful natural antioxidant and antibacterial agent for extending the shelf life and quality of fresh Pangasius fish balls in a refrigerator. Compared to the control and synthetic antioxidants (BHT and AA), the addition of MJPE considerably slowed pH rise, decreased cooking loss, maintained color characteristics and heme iron content, increased antioxidant activity, and inhibited lipid oxidation. Additionally, samples treated with MJPE showed decreased total viable counts during storage, suggesting enhanced microbiological stability. Higher MJPE concentrations T4 showed the most protective benefits of all the treatments. Overall, the results indicate that MJPE may be an appropriate clean-label substitute for artificial preservatives in fish products, enhancing their quality, safety, and stability during refrigeration. Future studies should focus on comprehensive chemical profiling to better understand the specific compounds responsible for the observed functional properties.

## Supporting information

S1 File S1 Dataset. (XLSX)
